# Supplementary material for: TGFβ signaling links early life endocrine-disrupting chemicals exposure to suppression of nucleotide excision repair in rat myometrial stem cells
Source: Cell Mol Life Sci. 2023 Sep 9;80(10):288. doi: 10.1007/s00018-023-04928-z (PMC10492698; doi:10.1007/s00018-023-04928-z)
Supplement: Supplementary file 1 — Supplementary file1 Supplemental Fig 1 Transcriptome and pathway enrichment analysis in EDC- over VEH-MMSCs. A) Volcano plot showing differentially expressed genes (DEGs) in EDC- over VEH-MMSCs. B) List of DEG belonging to TGFβ signaling. C) Pathway enrichment analysis in EDC-over VEH-MMSCs using the Hallmark MSigDB collection. The arrows indicate enriched pathways related to NER (orange) and TGFβ (red) signaling. D) List of DEG belonging to nucleotide excision repair (NER) pathway. FC: fold change (PDF 443 KB) [file 18_2023_4928_MOESM1_ESM.pdf]

A

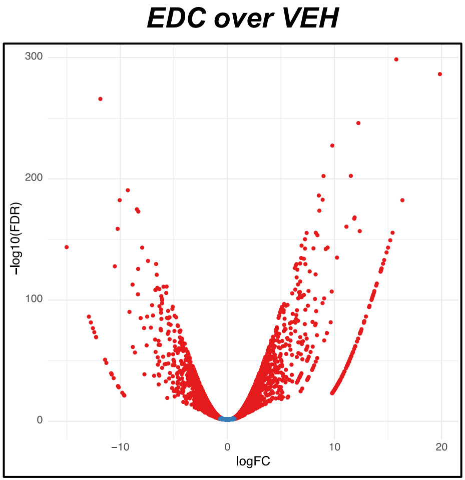

1487 up, 1455 down

B

	Gene	EDC_over_VEH
TGF-β signaling	Thbs2	12.24
	Inhbb	7.91
	Tgfbi	3.54
	Thbs1	2.34
	Gdf6	2.31
	Serpine1	1.61
	Pmepa1	1.48
	Ltbp2	1.16
	Ltbp1	1.12
	Inhba	1.12
	Junb	1.11
	Inha	-1.29
	Ltbp3	-1.49
	Ltbp4	-4.61

$Log_2 FC$

12

0

-4

C

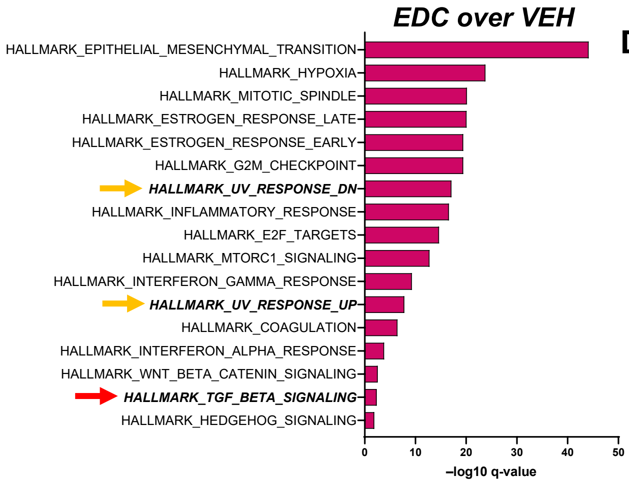

D

	Gene	EDC_over_VEH
NER signaling	Pole	1.17
	Polr2e	-1.01
	Xpc	-1.05
	Xpa	-1.25
	Pola2	-1.97
	Top2a	-2.32

$Log_2 FC$

12 0 -4
