# Supplementary figures and images for: TGFβ signaling links early life endocrine-disrupting chemicals exposure to suppression of nucleotide excision repair in rat myometrial stem cells
Source: Cell Mol Life Sci. 2023 Sep 9;80(10):288. doi: 10.1007/s00018-023-04928-z (PMC10492698; doi:10.1007/s00018-023-04928-z)

A

VEH+TGFβ1 over VEH

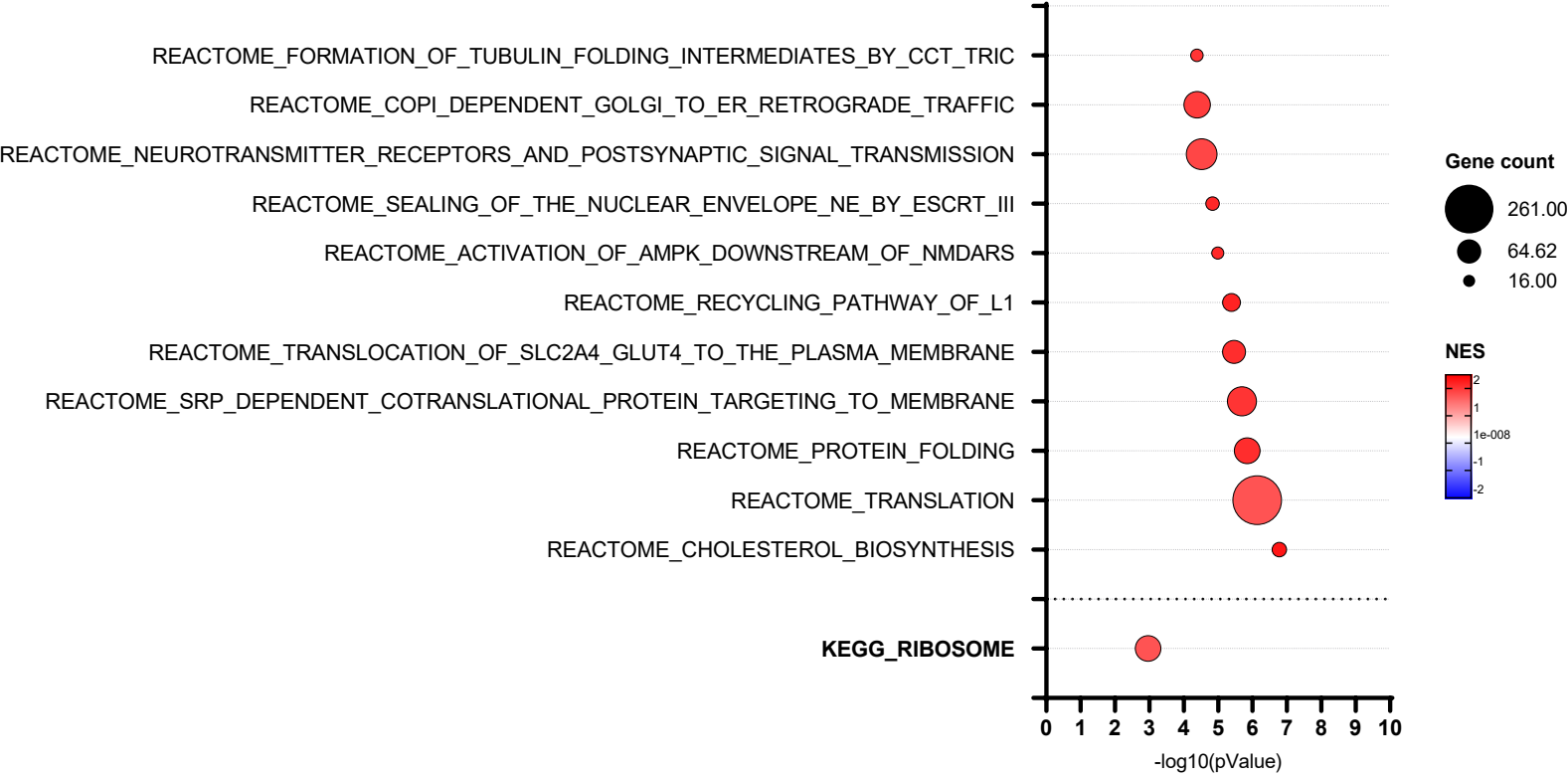

B

EDC+TGFβ RI inhibitor over EDC

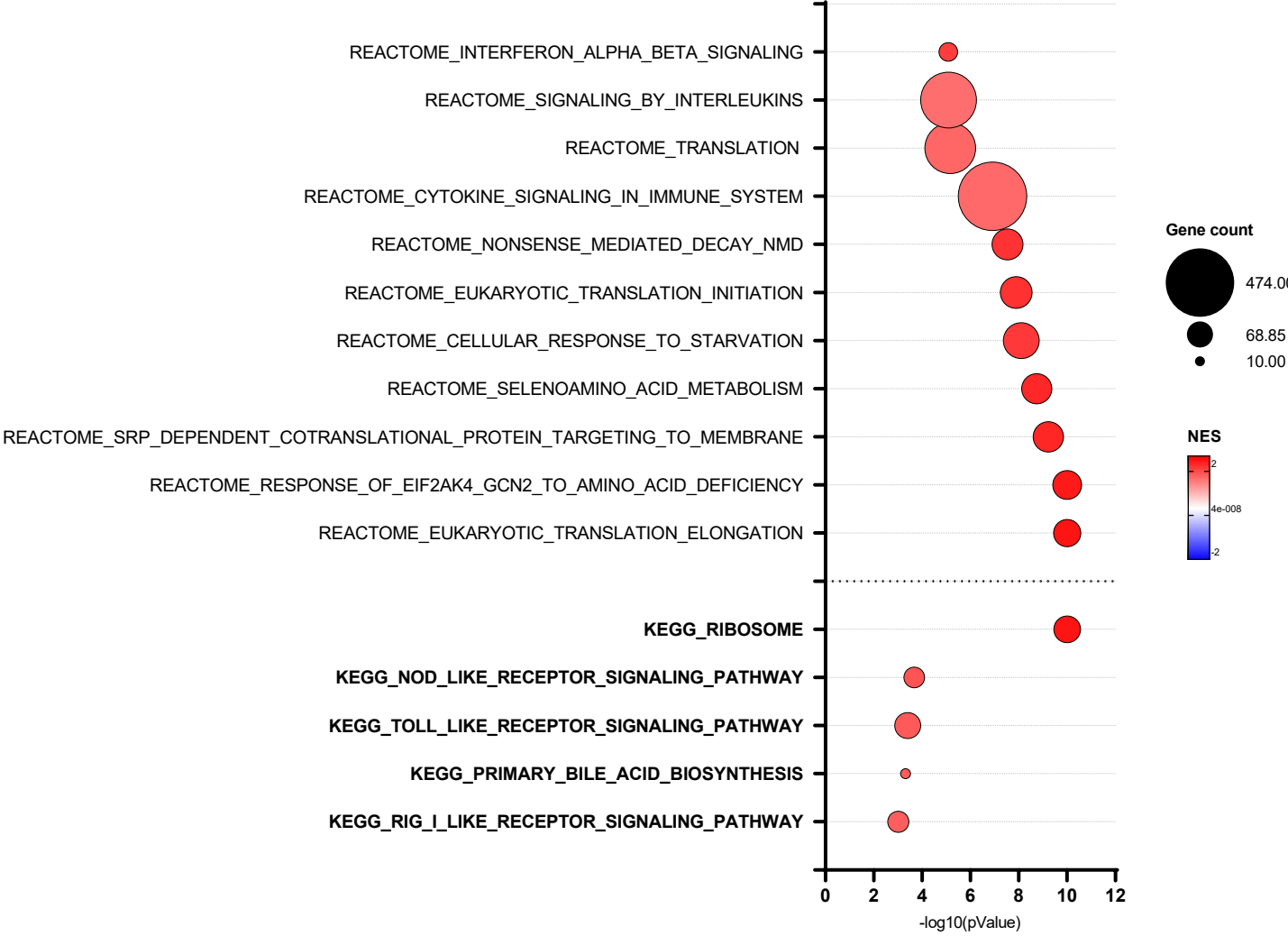

Supplement: Supplementary file 2 — Supplementary file2 Supplemental Fig 2 Effect of TGFβ1 pathway activation and inhibition on pathway enrichment of rat VEH- and EDC-MMSCs. A bubble chart of the Gene Set Enrichment Analysis (GSEA) using the RREACTOME and KEGG MSigDB collection for A) VEH-MMSCs treated with vehicle or TGFβ1, and B) EDC-MMSCs treated with vehicle or TGFβ Receptor I inhibitor comparisons. Normalized enrichment score (NES) is a metric whose sign corresponds to which end of the dataset is enriched in the tested gene set (PDF 125 KB) [file 18_2023_4928_MOESM2_ESM.pdf]
